# Reporter‐based forward genetic screen to identify bundle sheath anatomy mutants in *A. thaliana*

**DOI:** 10.1111/tpj.14165

**Published:** 2019-01-18

**Authors:** Florian Döring, Kumari Billakurthi, Udo Gowik, Stefanie Sultmanis, Roxana Khoshravesh, Shipan Das Gupta, Tammy L. Sage, Peter Westhoff

**Affiliations:** ^1^ Institute of Plant Molecular and Developmental Biology Heinrich‐Heine University Universitätsstrasse 1 40225 Duesseldorf Germany; ^2^ Cluster of Excellence on Plant Sciences ‘From Complex Traits towards Synthetic Modules’ 40225 Duesseldorf and 50923 Cologne Germany; ^3^ Department of Biology and Environmental Sciences Carl Von Ossietzky University Ammerlaender Heerstrasse 114 26129 Oldenburg Germany; ^4^ Department of Ecology and Evolutionary Biology The University of Toronto Toronto ON M5S 3B2 Canada

**Keywords:** C_4_ photosynthesis, EMS mutagenesis, bundle sheath cell, *Arabidopsis thaliana*, *GFP*, *LUC*, technical advance

## Abstract

The evolution of C_4_ photosynthesis proceeded stepwise with each small step increasing the fitness of the plant. An important pre‐condition for the introduction of a functional C_4_ cycle is the photosynthetic activation of the C_3_ bundle sheath by increasing its volume and organelle number. Therefore, to engineer C_4_ photosynthesis into existing C_3_ crops, information about genes that control the bundle sheath cell size and organelle content is needed. However, very little information is known about the genes that could be manipulated to create a more C_4_–like bundle sheath. To this end, an ethylmethanesulfonate (EMS)‐based forward genetic screen was established in the Brassicaceae C_3 _species *Arabidopsis thaliana*. To ensure a high‐throughput primary screen, the bundle sheath cells of *A. thaliana* were labeled using a luciferase (LUC68) or by a chloroplast‐targeted green fluorescent protein (sGFP) reporter using a bundle sheath specific promoter. The signal strengths of the reporter genes were used as a proxy to search for mutants with altered bundle sheath anatomy. Here, we show that our genetic screen predominantly identified mutants that were primarily affected in the architecture of the vascular bundle, and led to an increase in bundle sheath volume. By using a mapping‐by‐sequencing approach the genomic segments that contained mutated candidate genes were identified.

## Significance statement

We present a robust method using *Arabidopsis thaliana* as a model system to efficiently induce mutations that affect bundle sheath anatomy using ethyl methanesulfonate and to map the responsible genomic region by mapping by sequencing. The key feature of this method is the use of easily detectable reporter genes for the primary screening of mutant candidates. We believe that this method is generally applicable for creating mutants that are affected in inner leaf anatomy.

## Introduction

C_4_ plants surpass C_3_ species in their photosynthetic performance under conditions of high light, hot temperatures and drought (Ehleringer *et al*., 1991) due to their unique mode of photosynthesis that is characterized by a division of labor between two different cell leaf types, the mesophyll and, most commonly, the bundle sheath cells (Edwards and Voznesenskaya, 2011). These two cell types are arranged in a wreath‐like structure around the vasculature termed the Kranz anatomy (Haberlandt, 1904) and build a single integrated metabolic system (Hatch, 1987). Atmospheric CO_2_ is first fixed in the mesophyll cells by phosphoenolpyruvate carboxylase, an oxygen insensitive carboxylase. The resulting C_4_ acid is then transported into the bundle sheath cells where it is decarboxylated by one or a combination of NADP‐, NAD‐dependent malic enzyme and phosphoenolpyruvate carboxykinase (Furbank, 2011; Wang *et al*., 2014). The released CO_2_ is thereby concentrated at the site of ribulose‐1,5‐bisphosphate carboxylase/oxygenase and finally channeled into the Calvin−Benson cycle. Due to this CO_2_ pumping mechanism photorespiration is largely abolished, resulting in the superior photosynthetic efficiency of C_4_ plants (Zhu *et al*., 2010).

C_4_ photosynthesis occurs only in the angiosperms (Ehleringer *et al*., 1997) and has evolved independently more than 60 times (Sage *et al*., 2011; Sage, 2016). The polyphyletic origin of the C_4_ photosynthetic pathway indicates that, from the genetic point of view, it must have been relatively easy to evolve a C_4_ species from a C_3_ species. Indeed, quantitative modeling has shown that C_4_ evolution proceeded step by step and that each of these small evolutionary changes contributed to an increase in the general fitness of the plant (Heckmann *et al*., 2013).

Because of the high photosynthetic performance and the possible application of this knowledge in plant breeding, the molecular genetics and evolutionary basis of C_4_ photosynthesis has been studied intensively in the last decade (von Caemmerer *et al*., 2012). If existing C_3_ crops, such as rice or wheat, could be converted by genetic engineering to operate a C_4_ photosynthetic pathway, the predicted enhancement in photosynthetic efficiency could possibly be used to boost crop yields (Dawe, 2007; Zhu *et al*., 2010).

The lack of an appropriate C_4_‐like bundle sheath in C_3_ plants is one major obstacle that has to be overcome in this endeavor. While the bundle sheath in the C_4_ species is enlarged and rich in chloroplasts and mitochondria, the corresponding tissue in the C_3_ species is usually not very prominent and relatively poor in organelles (Sage *et al*., 2014). This finding indicates that this tissue does not play a major role in leaf photosynthesis of C_3_ plants (Kinsman and Pyke, 1998; Leegood, 2008). The exact physiological role of bundle sheath cells in C_3_ plants is currently not well understood. It is assumed that they function in phloem loading and unloading and contribute to the mechanical support of the leaf (Van Bel, 1993; Kinsman and Pyke, 1998; Griffiths *et al*., 2013). Transcript profiling of *Arabidopsis thaliana* bundle sheath cells has indicated that the bundle sheath tissue, at least in Brassicacean species, is highly active in sulfur and glucosinolate metabolism (Aubry *et al*., 2014). Cross‐species expression specificity of the bundle sheath‐specific promoter of the gene encoding the P‐subunit of glycine decarboxylase (*GLDPA*) from the Asteraceae C_4_ species *Flaveria trinervia* showed that bundle sheath‐specific expression was maintained in the C_3_ species *Arabidopsis thaliana* (Engelmann *et al*., 2008; Wiludda *et al*., 2012). Conversely, bundle sheath‐specific expression of the promoter of the sulfate transporter gene *SULTR2;2* from *A. thaliana* (Takahashi *et al*., 2000) maintained its bundle sheath specificity in the Asteraceae C_4_ species *Flaveria bidentis* (Kirschner *et al*., 2018). These findings indicated that the transcription‐regulatory system of bundle sheath cells, i.e. the interplay of *cis*‐regulatory elements with their cognate transcription factors, is at least partially conserved in dicotyledonous angiosperms and that a cryptic Kranz anatomy is already present in C_3_ species (Westhoff and Gowik, 2010).

Mutant analysis (Slewinski *et al*., 2012, 2014) and transcript profiling experiments (Wang *et al*., 2013) with maize indicated that the *SHORTROOT*‐*SCARECROW* transcriptional regulatory module (Sparks *et al*., 2016) is not only a key component in root patterning (Petricka *et al*., 2012), but also regulates the establishment of Kranz anatomy (Slewinski, 2013; Fouracre *et al*., 2014). In addition, the GOLDEN2‐like transcriptional regulator proteins play a role and can induce a C_3_ to C_4_ switch in bundle sheath characteristics (Wang *et al*., 2017).

Forward genetic screens have proved to be powerful and unbiased tools to dissect biological processes and identify their underlying genes and regulatory networks. Here, we present the design of a simple screening method on the tractable genetic model plant *A. thaliana*. The aim of this screen was to identify the bundle sheath developmental genes that are involved in the ontogeny and functional maintenance of the bundle sheath. These genes might be possible targets to increase the size and organelle number of this cell type. A successful forward genetic screen is defined by a high‐throughput, reliable and robust primary screen for mutants in which 1000s of plants have to be analyzed (Page and Grossniklaus, 2002). As the bundle sheath of *A. thaliana* is not very prominent and its cells contain only a few chloroplasts, we used reporter genes to label the bundle sheath or its chloroplasts. *Arabidopsis* lines that express these reporter genes should deliver an easy primary screen for mutants that were potentially affected in bundle sheath size or chloroplast numbers. To this end, we used the *GLDPA* promoter from *F. trinervia* that is active in the bundle sheath but not in the mesophyll of *A. thaliana* (Engelmann *et al*., 2008) to drive the expression of reporter genes that encode either firefly luciferase 68 (LUC68) or a chloroplast‐targeted green fluorescent protein (sGFP). In *A. thaliana* leaves, *GLDPA*_Ft_ promoter activity starts after 5–6 days of germination. *Arabidopsis* lines homozygous for the p*GLDPA*_Ft_::*LUC68* or the p*GLDPA*_Ft_::TP_*RbcS* –_ *sGFP* reporter genes were generated and mutagenized with the chemical mutagen ethyl methanesulfonate (EMS). The level of reporter gene expression served as a proxy to select mutants with altered bundle sheath anatomy. Mutant lines that contained an intact reporter gene and whose reporter expression deviated strongly from the non‐mutagenized reference lines were then further analyzed by light and electron microscopy for alterations in bundle sheath anatomy.

## Results

### Design of the mutant screen

**Figure 1:**
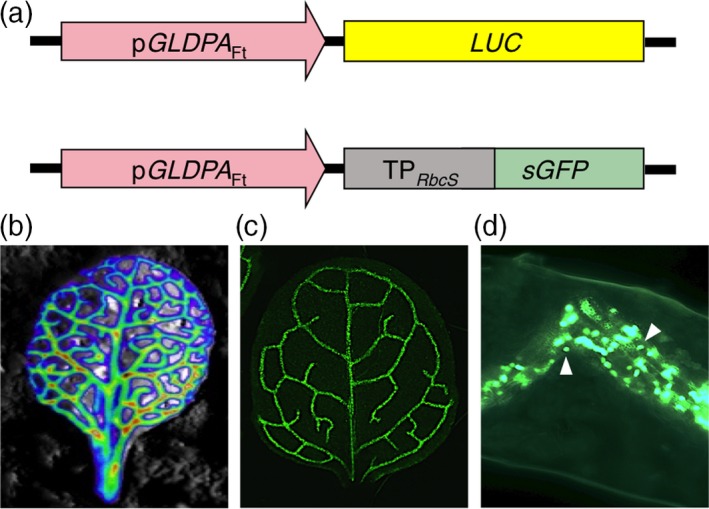
Labeling the bundle sheath of leaves of *Arabidopsis thaliana* using luciferase (*LUC*) and green fluorescent protein (*GFP*) reporter genes, respectively, which were driven by the promoter of the gene encoding the P‐subunit of glycine decarboxylase of the C_4_ plant *Flaveria trinervi*a (p*GLDPA*_F__t_). (a) The constructs used to generate the p*GLDPA*_Ft_−*LUC* and p*GLDPA*_Ft_−*GFP* reference lines. (b) Luminescence of a leaf of the *LUC* reference line. (c) GFP fluorescence of a leaf of the *GFP* reference line. (d) Longitudinal section of veins of the reference line showing GFP fluorescence localized in the chloroplasts (arrowheads) of the bundle sheath and vasculature.

This study aimed at designing a non‐destructive and robust screen to quickly and reliably identify mutant plants with altered bundle sheath anatomy, i.e. mutants that were affected in bundle sheath cell size and/or chloroplast numbers or volume within the bundle sheath cells. To this end, the bundle sheath cells of *A. thaliana* (Ecotype Columbia‐0) were labeled by a reporter gene that allowed non‐destructive and large‐scale phenotyping of segregating M2 populations. We generated *LUC* and *GFP* reporter lines (Figure 1), in which *A. thaliana* bundle sheath cells and vascular tissue were labeled with their respective reporter genes by expressing these under the control of the 1571‐bp 5′‐flanking region of the glycine decarboxylase P protein gene (*GLDPA*) of C_4_ Asteraceae species *F. trinervia* (Figure 1a–c). This promoter region is specifically and highly active in the bundle sheath cells and vascular tissue of *A. thaliana* (Engelmann *et al*., 2008; Wiludda *et al*., 2012). Moreover, the GFP protein in the *GFP* reporter gene line was targeted to the chloroplasts of the bundle sheath and vascular tissue cells by adding a highly active and well characterized transit peptide of Rubisco small subunit (Kim *et al*., 2010) to the gene. This allowed the ability to differentiate between single chloroplasts within the cells and is shown in leaf transverse sections (Figure 1d). In general, both reporter lines should allow quick and easy detection of changes in the bundle sheath cell anatomy, i.e. cell size based on the diameter of the bundle sheath strand. Additionally, the *GFP* signal intensity might be correlated with chloroplast numbers and/or volume of the bundle sheath cells, as each chloroplast is labeled with the GFP protein. Therefore, in both the LUC and GFP reporter gene‐based screens, mutant lines were primarily selected with respect to deviations in the reporter protein signal activity, as assessed by an imaging system (LUC) or light microscopy (GFP).

### EMS‐based genetic screen with bundle sheath‐labeled reporter gene lines

Approximately 160 000 seeds were mutagenized with EMS (40 000 *LUC* reporter gene line seeds; 120 000 *GFP* reporter gene line seeds) and sown in soil in large trays under greenhouse conditions. A survival rate of about 50% was observed in the M1 generation and seeds were harvested in pools of 30–50 plants from the remaining 80 000 M1 plants. Approximately 45 000 M1 plants were needed under the given EMS concentration to have a 95% chance of exploring a mutation in any given G:C base pair (Jander *et al*., 2003). Therefore, a saturating EMS screen was reached by mutagenizing most G:C base pairs in the genome of *A. thaliana*. We expected the number of mutations per genome to be randomly distributed, following a Poisson distribution and calculated approximately one embryonic‐lethal mutation per mutagenized genome (Pollock and Larkin, 2004). In addition, 2.2% of the plants in the M2 generation displayed a pale chlorophyll phenotype, therefore the EMS treatment could be considered as a success (Kim *et al*., 2006).

**Figure 2:**
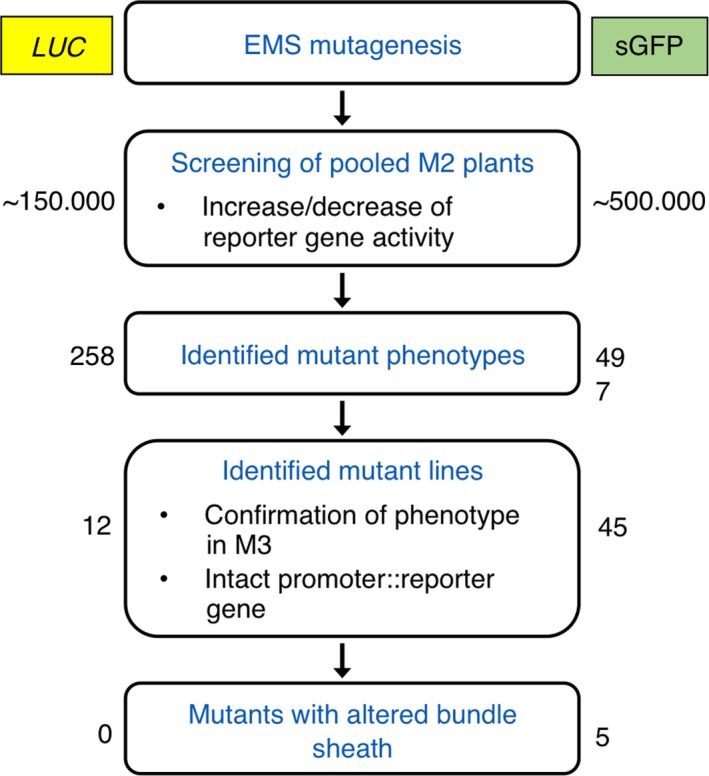
Workflow of the EMS screen with both reporter gene lines (*LUC* and *GFP*). Number of plants/mutants of each step of the mutant screen using the *LUC* and *GFP* reporter gene lines are shown on the left side and right side, respectively.

The general workflow of the EMS‐based genetic screen, which aims at identifying mutants altered in bundle sheath anatomy and function, is depicted in Figure 2. Seeds from each M2 pool were sown individually on large trays in the greenhouse, and single leaves or whole seedlings were screened for aberrant reporter gene expression (e.g. stronger or weaker reporter gene signal in the bundle sheath). In total, 755 primary mutants were identified; 258 mutants with a *LUC* background and 497 mutants with a *GFP* background. The phenotype of each mutant line was assessed in the following M3 generation for its stability, whereby only mutant lines with a strong deviation in reporter gene signal intensity compared with the reference line were selected. In total, 85 mutant lines with an *LUC* background and 145 mutants with a *GFP* background remained. Furthermore, complete reporter gene constructs (p*GLDPA*_Ft_::*LUC* and p*GLDPA*_Ft_::*TP*_*RbcS*_ ‐ s*GFP*) were amplified via polymerase chain reaction (PCR) and sequenced in the background of every identified mutant to exclude aberrant phenotypes that were based on mutations within the promoter reporter gene construct. Almost 75% of the mutant lines had to be discarded due to mutations in the promoter reporter gene construct. Most of these contained mutations only in the reporter gene sequence except two mutant lines that possessed mutations in both the promoter and the reporter gene sequence. There were no mutant lines with mutations only in the promoter region. Nevertheless, 12 mutant lines with the *LUC* reporter gene and 45 mutant lines with the *GFP* reporter gene were both stable and contained intact reporter genes.

**Figure 3:**
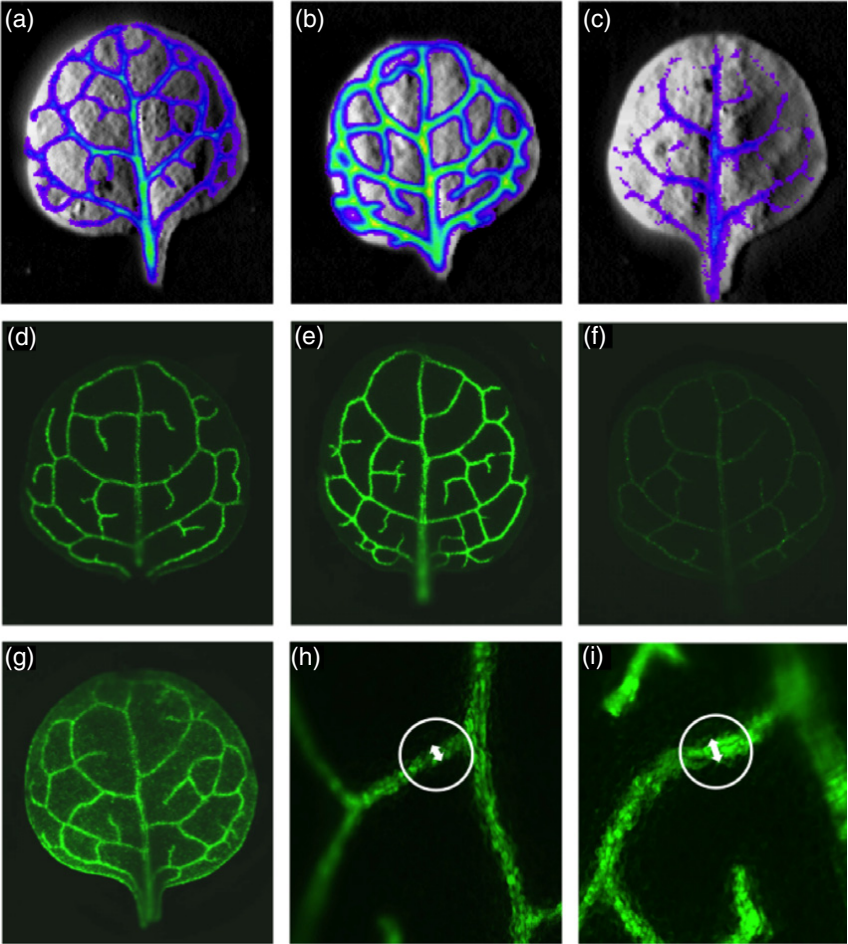
Results of the EMS primary screen. (a) Luminescence signal emitted by the reference line compared with (b) a mutant line with increased and (c) decreased reporter gene signal relative to control/reference line. (d) GFP fluorescence of the reference line compared with (e–g) three different classes of primary mutants with either (e) more, (f) less, or (g) a diffused reporter gene activity in the leaves. (h, i) A close‐up view of 3° veins of (h) the reference line and (i) mutant line G‐19. The width of the veins is emphasized by white arrows.

The EMS‐based *LUC* reporter screen resulted in six mutant lines with increased and six mutant lines with decreased reporter gene activity (Figure 3b and c), while the *GFP* reporter screen resulted in 22 and 19 lines with increased and decreased reporter gene activity relative to control/reference lines, respectively (Figure 3e, f). Moreover, four mutant lines possessed a diffused GFP signal in which the reporter gene signal was clearly detectable in the mesophyll tissue (Figure 3g). Figure S1 shows the relative LUC and GFP signal intensity of all mutant lines. Some of the mutants with a diffused GFP signal were kept for further analyses as the loss in tissue‐specificity of our reporter gene might be linked to altered bundle sheath or mesophyll development or to mutations in genes affecting the transcription and/or post‐transcriptional regulation of the p*GLDPA*_*Ft*_ promoter (Engelmann *et al*., 2008; Wiludda *et al*., 2012). Intriguingly, seven mutants with increased GFP signal intensity also contained bundle sheath strands (vascular tissue plus bundle sheath) with an increase in diameter in comparison with the reference line (Figure 3h, i), which might be caused by an increase in either the vascular tissue or bundle sheath tissue, or a combination of both.

### Microscopic analysis of EMS‐generated mutant lines

Our primary screening criterion was based on the reporter gene activity and, therefore, we could not clearly assign changes in reporter gene expression to anatomical alterations of bundle sheath cells in identified mutants. To address this question, we selected 27 mutant lines from the primary screen with the strongest phenotypes in terms of signal intensity and width of the bundle sheath strands (G01–G25 and L01, L02) (Table S1) to image 1.5 μ thin sections of resin embedded leaf tissue with the light microscope. Among this subset of mutant lines, 20 mutant lines possessed an increased GFP signal, three mutant lines showed less GFP signal, and two mutant lines exhibited a diffused GFP reporter signal. In addition, two mutant lines of the *LUC* reporter screen with an increased reporter signal were included in the study.

**Figure 4:**
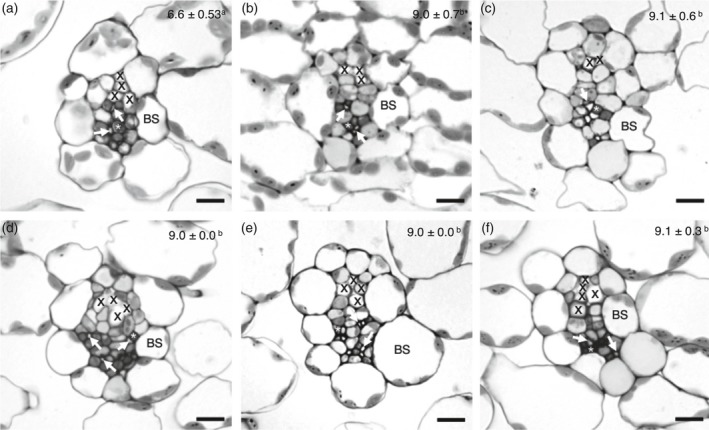
Light micrographs illustrating cross‐sections of a third‐order vein. (a) Reference line. (b) Line 14. (c) Line 15. (d) Line 17. (e) Line 19. (f) Line 20. Mean number of bundle sheath cells surrounding the vascular tissue is depicted at the upper right corner of the respective line. Values marked by different letters are significantly different (*P* < 0.05) as determined from Kruskal–Wallis one‐way analysis of variance on ranks with pair‐wise multiple comparison procedures (Tukey Test). Statistical analysis was run using Sigma‐plot version 12.5. H = 33.008 with 5 degrees of freedom. Bar, 10 μm. BS, bundle sheath; X, vessel element; * marks companion cell; white arrow marks sieve tube element.

Transverse sections of each replicate were compared with those of the reference line, and the 3° higher‐order veins were analyzed with respect to the anatomy of the bundle sheath and vascular tissue. Five mutant lines (G14, G15, G17, G19 and G20) were identified, whose bundle sheath tissue contained more cells in the radial direction as compared with the reference line (Figure 4a versus Figure 4b–f), although no differences could be found in paradermal view (Figure S2). The bundle sheath cell number was determined by counting the number of bundle sheath cells that surrounded the vascular tissue of third order veins. From each line, three biological replicates were investigated and, from each replicate, three different third order veins were investigated. Additionally, the mutant lines G14, G15, G17 and G19 showed an increased number of chloroplast‐containing cells within the phloem tissue (companion cell and vascular parenchyma; Maeda *et al*., 2008) as well as an increase in sieve elements relative to the reference line (Figure 4a versus Figure b–e). Mutant line G20 showed an amplification of the tracheary elements (Figure 4f).

Due to expected chloroplast targeting of the *GFP* reporter, we hypothesized that changes in the GFP signal intensity in identified mutant lines might derive from increased chloroplast number, size, or structure. However, further analyses of selected mutant lines revealed that none indicated an obvious difference in chloroplast number or sizes in bundle sheath cells.

Analyses of all chloroplast‐containing cells of nine mutant lines (G10, G13, G14, G18, G19, G20, G22, G23, and G25) and the reference line with transmission electron microscopy (TEM) indicated no obvious changes in chloroplast ultrastructure with the exception of line G19. In this line, the majority of mesophyll and bundle sheath cell chloroplasts contained prominent nucleoids and two to four grana with extensive stacking of long thylakoids (Figure S3e, g−j). This phenotype was more prominent in mesophyll cells. The lumens of the grana thylakoids in line G19 were narrow and disorganized relative to the reference line (Figure S3j versus Figure S3i). Some chloroplasts also contained numerous vesicles (Figure S3h). Numerous prominent nucleoids that contain chloroplast DNA and plastid nucleoid associated proteins (Powikrowska *et al*., 2014) were associated with grana and vesicles (Figure S3e, g, h, j).

### Leaf morphology and growth characteristics of mutant lines G14, G15, G17, G19, and G20

**Figure 5:**
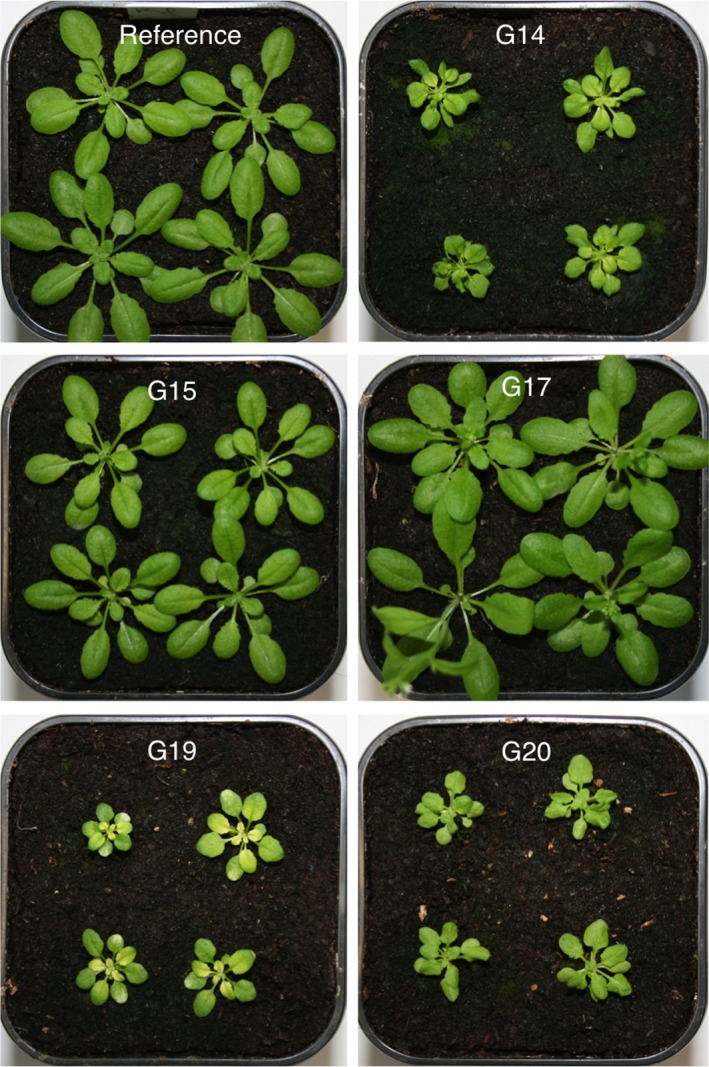
Overview of mutant lines G14, G15, G17, G19, G20, and the reference line. All plants were 28 days old.

Mutant lines G14, G19, and G20 showed impaired leaf morphology and growth characteristics relative to the reference line. The growth of the mutant lines was strongly reduced and their leaves were smaller in size compared with those of the reference line. Furthermore, in mutant line G20 the first leaf pair, but not the cotyledons, displayed a partial reticulate leaf pattern, i.e. there were prominent, green bundles on a pale lamina. These green bundles on a pale lamina were specific to the tip of the leaf, whereas the lamina of the leaf base remained mostly green. All other leaves did not develop this reticulated leaf phenotype, however we observed slightly pale leaves in general, especially in the emerging leaves (Figure 5).

### Mapping of the EMS‐induced point mutations within the genome

To identify affected genes causing the mutant phenotypes a mapping‐by‐sequencing approach (Schneeberger *et al*., 2009) was conducted. However, we could not follow the standard procedures for gene mapping/identification using outcross populations because we depended on reporter gene expression in the bundle sheath to identify the individual mutant phenotype in the segregating mapping population. It has been shown before that the use of backcross populations results in sufficient genetic diversity to identify the causative point mutation (Abe *et al*., 2012; James *et al*., 2013). Therefore, we backcrossed our mutant lines with the non‐mutagenized reference line. F1 plants were propagated, resulting in the F2 backcross population, which showed a 3:1 segregation of the recessive mutant phenotype according to Mendelian law.

**Figure 6:**
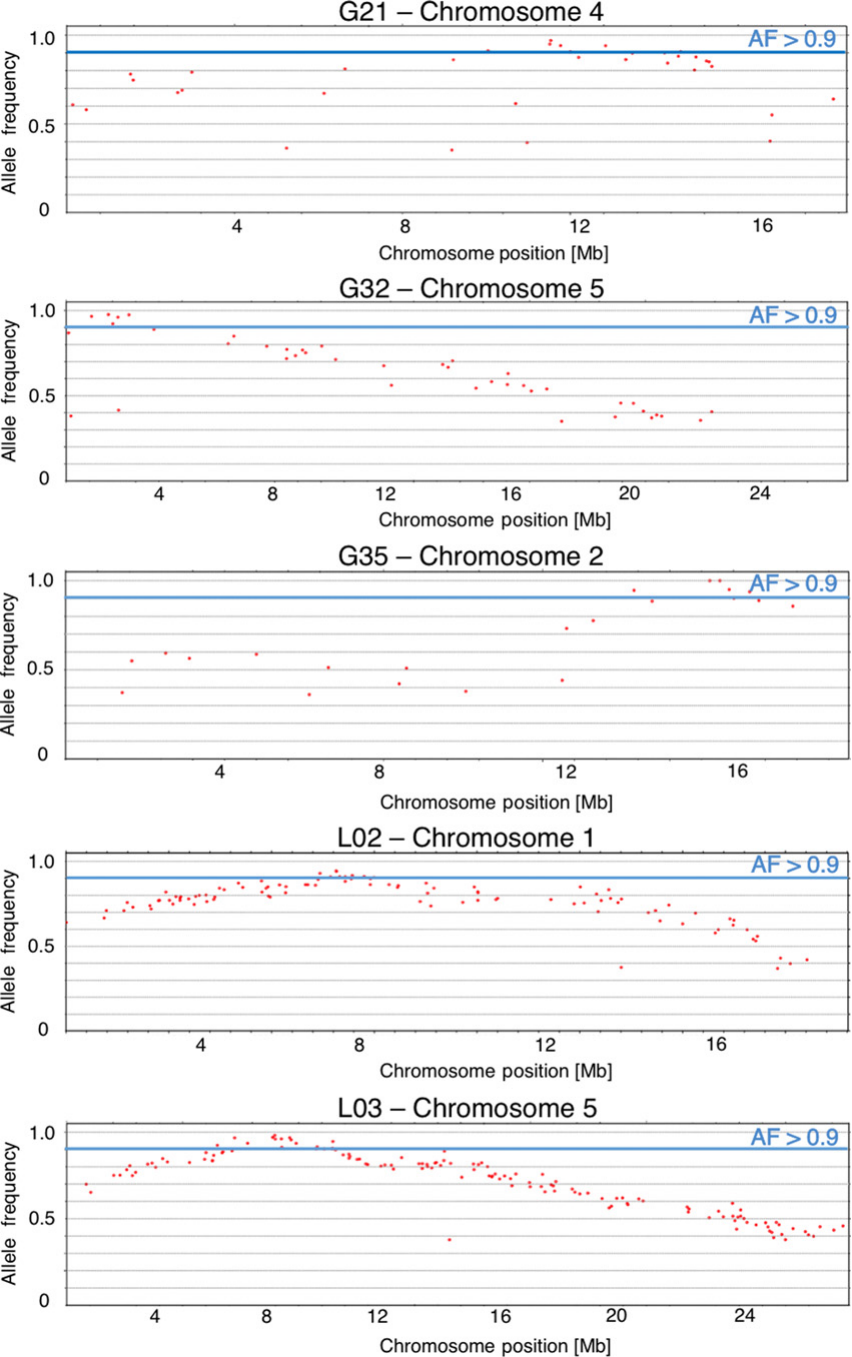
Allelic frequencies for mutant lines G21, G32, G35, L02 and L03. Allelic frequencies (AF) for all SNPs resolved using the reporter gene line parent and BCF2 mutant whole genome sequence data. Genes containing a non‐synonymous SNP with AF >0.9 were considered as candidate genes. Only the chromosome with allelic frequencies >0.9 is shown for each mutant line.

To obtain a proof of concept of our mapping strategy, the first five homozygous EMS‐generated mutant lines G21, G32, G35, L02 and L03 were selected for bulked segregant analyses and studied in parallel with light and electron microscopy described above. Neighboring EMS‐induced point mutations close to the causable single nucleotide polymorphism (SNP)/the causative mutation were expected to be in linkage disequilibrium and, therefore, should not recombine. We analyzed the sequencing data of the bulked homozygous mutant plants by using a SHOREmap backcross scheme (Sun and Schneeberger, 2015) by which the peak of the confidence interval was mapped by analyzing the allelic frequencies (AFs) of the EMS‐induced SNPs. By pursuing this approach, clear candidate regions (AF > 0.9) could be identified in all five mutants (Figures 6 and S4a−c). Between two and 10 mutations altering the coding region (exons) or the splicing sites of the protein encoding genes could be identified for each of these mutant lines. The candidate genes will be analyzed by targeted gene knock‐out experiments at a later date.

## Discussion

The photosynthetic activation of the bundle sheath, which is characterized by an increase in cell size and chloroplast volume, is considered to be a key step in the evolution towards C_4_ photosynthesis (Westhoff and Gowik, 2010; Sage *et al*., 2014). With the exception of the SCARECROW/SHORT‐ROOT (SCR/SHR) and Golden*2*‐like (GLK1/GLK2) transcription factors, our knowledge on the gene regulatory networks that are additionally involved in the activation of the bundle sheath is rather poor. (Rossini *et al*., 2001; Slewinski *et al*., 2012, 2014; Cui *et al*., 2014; Wang *et al*., 2017). To this end, we developed a forward genetic mutant screen using *A. thaliana* aiming to identify mutants with an increased bundle sheath cell size and/or increased numbers of chloroplasts within the bundle sheath cells.

Both reporter gene lines, *LUC* and *GFP* were subjected to EMS mutagenesis and the primary screen of M2 plants was performed in parallel. For the primary screen, the reporter signal intensity was used as a proxy, whereby M2 mutant plants with deviating reporter gene activity were selected (Figure 3). The *GFP* reporter gene turned out to be better suited for a high‐throughput screen than the *LUC* reporter gene. *LUC* reporter plants required an extra incubation step of leaves with the substrate d‐Luciferin to generate the luminescence signal. In total, we could screen twice as many plants at a given time with the *GFP* reporter compared with the *LUC* reporter. Furthermore, the spatial resolution of the reporter gene signal in the primary screen was higher in *GFP* plants in comparison with *LUC* reporter plants (Figures 1 and 3). Therefore, we exclusively continued the screening process with the *GFP* reporter line from the early screening stages.

Out of the 755 identified primary mutants, more than 93% had to be eliminated due to instability of the phenotype or point mutations within the reporter gene construct (Figure 2). After sorting out these lines, we were still left with a reasonable number of 57 mutant lines (12 *LUC* and 45 *GFP*) (Figure 2). Furthermore, the byproducts of this genetic screen such as mutant lines with mutations in the 1571‐bp 5′‐flanking region of the *GLDPA* gene could be helpful in elucidating the regulation of this complex 5′ flanking segments with respect to the balance of transcriptional versus post‐transcriptional gene control (Engelmann *et al*., 2008; Wiludda *et al*., 2012).

To investigate the correlation of altered reporter gene expression with an altered bundle sheath anatomy, 27 mutant lines (25 from the *GFP*‐based screen and two from the *LUC* experiments) with strong deviations in reporter gene activity were used for light microscopic analysis (Table S1). Out of these 27 mutant lines, five mutant lines (G14, G15, G17, G19 and G20) showed altered bundle sheath structure. All of the five mutants possessed more bundle sheath cells, which were accompanied by an apparent increase in vascular tissue per vein (Figure 4).

We did not find any mutant with either increased chloroplast number per bundle sheath cell or altered chloroplast size, although the five mutant lines mentioned above exhibited a strong increase in GFP fluorescence which, due to the RbcS transit peptide, was localized in the chloroplasts (Figure S1). This enhanced GFP signal could be derived either by an increase in xylem and/or phloem parenchyma and companion cells, all of which contain chloroplasts and/or could be explained by an increased number of bundle sheath cells per se in the mutant lines (Figure 4).

Mutant line G19 showed deviations in the stacking of thylakoids and the accumulation of nucleoids (Figure S3). Regulation of thylakoid organization/stacking is controlled by numerous factors (Gao *et al*., 2006; Armbruster *et al*., 2013; Pribil *et al*., 2014). Among those, CURT1 proteins are involved in bringing about membrane curvature at the grana margins, and increased amounts of CURT1 proteins give rise to grana with a large number of thylakoids (Armbruster *et al*., 2013). Thylakoid formation is also linked with nucleoid distribution (Kobayashi *et al*., 2013) and the spatial relationship is important for the assembly of the photosynthetic apparatus (Powikrowska *et al*., 2014). Isolation of the gene responsible for the phenotype of mutant G19 may identify an additional genetic factor regulating thylakoid biogenesis.

All the five mutants harbored an enlarged bundle sheath compartment. This enlargement was not caused by an increase in the bundle sheath cell sizes, but rather originated from the increase of bundle sheath cell numbers. In summary, almost 20% of the mutant lines (5/27) of which an altered reporter gene signal was detected in the primary screen could be clearly linked to anatomical changes within the bundle sheath and/or the vascular tissue. In the remaining mutant lines with no obvious aberration in bundle sheath/vascular tissue anatomy the increase/decrease of reporter gene signal might be caused by a transcriptional or translational perturbation of reporter gene expression. Therefore, we conclude that our screening strategy, i.e. using the activity of a reporter gene driven by a tissue‐specific promoter as a rapid proxy in the primary screen was successful in identifying mutants affected in the anatomy of the bundle and its sheath.

As stated above, the EMS mutant screen did not result in any mutant lines that were exclusively affected in bundle sheath anatomy. The increase in bundle sheath cell number was always associated with an expansion of the vascular tissue, probably due to enhanced cell division within the vascular tissue. The ontogenetic relation of the vascular bundle and the surrounding bundle sheath layer is already well described in grasses (Dengler *et al*., 1985; Bosabalidis *et al*., 1994). All C_3_ grasses as well as many C_4_ grasses develop a double sheath, i.e. the vascular tissue is encircled by a mestome sheath that itself is enclosed by a layer of parenchymatous sheath cells. By contrast, C_4_ grasses of the NADP‐malic enzyme subtype have a single sheath and do not have a mestome sheath (Brown, 1975; Hattersley and Watson, 1976; Rao and Dixon, 2016). Dengler *et al*. (1985) provided a detailed study on the origin of the bundle sheath in single‐sheath C_4_ and double‐sheath C_4_ and C_3_ grasses. They reported that that the vasculature and its adjacent cell layer were clonally related and derived from procambial initials in both double‐sheath (*Panicum effusum*,* Eleusine coracana* and *Sporobolus elongatus*) and single‐sheath C_4_ species (*Panicum bulbosum*,* Digitaria brownii* and *Cymbopogon procerus*). Nevertheless, it is not completely understood whether this situation is also true for minor veins. However, further studies in maize reported that both major and minor veins and the associated bundle sheath cells are derived from a single‐cell lineage in the median layer of the leaf primordium (Bosabalidis *et al*., 1994).

By contrast with grasses, our knowledge on the ontogeny of the bundle sheath in dicots is limited. It has been reported that the bundle sheaths of C_3_ and C_4_ *Cleome* species, originate from more than one layer of ground meristem cells and only adaxial bundle sheath cells are of procambial origin (Koteyeva *et al*., 2014). As the bundle sheath and vascular tissue either completely or partially arise from the same cell lineage, changes in the vascular tissue might subsequently result in changes in the bundle sheath anatomy as well. Moreover, the *GLDPA* promoter of the C_4_ species *Flaveria trinervia,* which was used to drive the reporter gene expression in this study, contributes to the mutant characteristics and phenotypic spectrum obtained. The *GLDPA*_Ft_ promoter is highly active in both the bundle sheath and the vascular tissue of *Arabidopsis* (Engelmann *et al*., 2008; Wiludda *et al*., 2012). Hence, the use of this promoter inevitably produced mutants primarily affected in vascular tissue. Therefore, the use of an alternative bundle sheath‐specific promoter for a mutagenesis screen might result in mutants affected only in bundle sheath anatomy. A promoter that drives expression exclusively in the bundle sheath of *Arabidopsis* is yet to be identified as all the bundle sheath promoters known for dicots, in varying degrees, are also active in the vasculature (Engelmann *et al*., 2008; Kirschner *et al*., 2018). Alternatively, the specificity problem could be overcome by additional labeling of the vascular tissue with a second reporter gene. This two‐reporter gene system would help to separate mutants only affected in the bundle sheath from mutants affected both in the bundle sheath and the vasculature. The promoters of the *SHORT‐ROOT* (*SHR*), *Sultr2;1* and *SWEET1* genes of *Arabidopsis* that encode a GRAS family transcription factor, a sulfate transporter and a sucrose efflux transporter, respectively, are specifically active in the vasculature of developed *Arabidopsis* leaves and might serve as suitable candidates to additionally label the vasculature (Takahashi *et al*., 2000; Chen *et al*., 2012; Cui *et al*., 2014).

Although our screen did not directly deliver the type of mutants we were aiming for, the mutants obtained should nevertheless be helpful in understanding the ontogeny of the bundle sheath in the context of vascular tissue. In *Arabidopsis*, vascular cell proliferation and balance of xylem and phloem tissue production within a vascular strand is controlled by numerous factors (Schuetz *et al*., 2013; Furuta *et al*., 2014). Our screening strategy might therefore be a straightforward approach in identifying genes that are primarily involved in the differentiation of the vasculature and its ontogeny.

A successful forward genetic approach requires that the mutant genes identified can be molecularly identified, i.e. the causative genes have to be mapped precisely to facilitate their identification and verification by state‐of‐the‐art tools such as gene knock‐out or replacement by the CRISPR/Cas9 technology (Hahn *et al*., 2017). By using a backcross procedure combined with bulk whole genome sequencing of F2 mutant plants, we were able to locate the causative mutations in an interval of 0.2–3.4 Mbp containing 2–10 mutated candidate genes. This finding is suitable for inducing CRISPR/Cas9‐mediated knock‐outs of the candidate genes. Moreover, the mapping resolution may be improved by enlarging the mapping population and therefore increasing the numbers of pooled F2 mutant plants for bulk sequencing (James *et al*., 2013). Our forward genetic approach relied on the use of reporter genes for the rapid and easy identification of mutant candidates in a primary proxy screen. It was coupled with a powerful mapping by sequencing strategy. We believe that this combination is very useful, if high‐throughput phenotyping of structural deviations at the cellular or tissue level is not possible.

## Experimental procedures

### Plant material

*Arabidopsis thaliana* (Ecotype Columbia‐0) was used as a genetic background for both reporter gene lines. Plants were grown under greenhouse conditions with supplementary light for 14 h per day at a photon flux density (PFD) of ~300 μmol m^−2^ s^−1^ or in climate chambers operated at 16 h light/8 h of darkness periods (~60 μmol m^−2^ sec^−1^) and a constant temperature of 21–22°C. The seeds were surface sterilized with bleach containing 20% Dan Klorix (Colgate‐Palmolive, Hamburg, Germany) and 0.02% Triton X‐100 for 5 min and washed four times with sterile water. After sterilization, the seeds were stratified at 4°C in the dark for at least 48 h before sowing in either soil (Floraton 1, Floragard, Oldenburg, Germany) or Petri dishes with half‐strength Murashige and Skoog (½MS) medium containing 0.6% agar and 1% sucrose.

### Generation of reporter gene lines

The pGreen Gateway vector containing the firefly luciferase 68 gene (pGreen‐*LUC68*) served as a backbone for the luciferase (*LUC*) reporter construct and was kindly provided by Franziska Turck (Adrian *et al*., 2010). The 1571‐bp 5′‐flanking region of the glycine decarboxylase P protein gene (*GLDPA*) of the C_4_ Asteraceae species *Flaveria trinervia* was amplified using PCR from a *GLDPA*_Ft_−GUS template (Engelmann *et al*., 2008) with specific oligonucleotides listed in Table S2 that added *att*B1 and *att*B2 sites to the PCR product. To introduce the *GLDPA*_*Ft*_ promoter sequence (p*GLDPA*_Ft_) into the Gateway entry vector pDONR221 the BP Clonase reaction (Gateway^®^ BP Clonase^®^ enzyme mix, ThermoFisher Scientific) was carried out as described by the manufacturer. The resulting pENTRY221‐p*GLDPA*_Ft_ was subsequently used for the LR Clonase reaction (Gateway^®^ LR Clonase^®^ enzyme mix, ThermoFisher Scientific) to transfer p*GLDPA*_Ft_ into pGreen−*LUC68* (pGreen−p*GLDPA*_Ft_::*LUC68*).

The binary plant transformation vector pBI121 (Clontech laboratories, Mountain View, CA, USA; Jefferson *et al*., 1987) was used to assemble the *GFP* reporter gene construct that included the p*GLDPA*_Ft_ region (Engelmann *et al*., 2008), the transit peptide sequence of the gene encoding the small subunit of ribulose‐1,5‐bisphosphate carboxylase/oxygenase of *A. thaliana* (AT1G67090; TP_*RbcS*_; Kim *et al*., 2010) and the s*GFP* gene sequence fused in frame with the TP_*RbcS*_ segment by using standard cloning procedures. The resulting final reporter gene construct was named p*GLDPA*_Ft_::*TP*_*RbcS*_−s*GFP*.

### Transformation of *A. thaliana*

Both reporter gene constructs were transferred into the *Agrobacterium tumefaciens* strain AGL1 (Lazo *et al*., 1991) by electroporation, and subsequently transformed into *A. thaliana* (Ecotype Columbia‐0) using the floral dip method (Logemann *et al*., 2006). T1 plants containing an intact reporter gene were first selected using kanamycin resistance followed by PCR amplification and sequencing of the entire reporter gene construct. Positive lines were propagated into the T3 generation, and homozygous plants were selected for the mutant screens.

### Ethyl methanesulfonate (EMS) mutagenesis

Approximately 40 000 seeds (~1.6 g) of the p*GLDPA*_Ft_::*LUC* reporter gene line and 120 000 (~4.8 g) seeds of the p*GLDPA*_Ft_::TP_*RbcS*_−*GFP* reporter gene line were used for EMS mutagenesis. The seeds were initially washed with 0.1% (v/v) Tween® 20 for 15 min, after which EMS (Sigma‐Aldrich, St. Louis, MO, USA) was added to a final concentration of 0.25% (v/v). The mixture was incubated for 16 h on a rotating platform at room temperature in the dark. Subsequently, the seeds were washed four times with sterile water, incubated again for 1 h on a rotating platform, and washed one last time in sterile water. After 2 days at 4°C, M1 seeds were sown evenly in soil. M2 seeds were harvested from a pool of 30–50 M1 plants. M2 plants were grown for about 14–17 days and used for the mutagenesis screen described below.

### Mutant screen

The first leaf pair was analyzed for both *LUC* and *GFP* aberrant reporter gene expression. In general, plants with more, less, or diffused reporter gene signal were selected at this point. The screen for LUC activity was performed with the imaging system Night Owl LB983 NC100 U (Berthold Technologies, Bad Wildbad, Germany) using the *in vivo* imaging software indiGO (Berthold Technologies, Bad Wildbad, Germany). Before screening, leaves were incubated in a 1 mm luciferin solution for 5 min after which LUC activity was detected (exposure time: 120 sec). The resultant signal in the bundle sheath/vasculature of the EMS‐mutagenized M2 populations was compared with the non‐mutagenized reporter line. In terms of the mutant screen with the *GFP* reporter gene line, plants of the M2 generation were screened for aberrant GFP expression under a fluorescence binocular microscope (Nikon SMZ25, Duesseldorf, Germany). All primary mutants selected at the M2 stage were analyzed again at the M3 stage, to confirm the aberrant mutant phenotype. Additionally signal intensity was measured for whole leaves and normalized to the leaf area using ImageJ (Schneider *et al*., 2012). Only mutant lines with at least 30% stronger or weaker signal intensities in the whole leaf were selected for further studies.

DNA was isolated from the mutant lines to check for point mutations in the reporter gene construct. The complete region (p*GLDPA*_Ft_::TP_*RbcS*_‐sGFP or p*GLDPA*_Ft_::*LUC68*) was amplified by PCR using the Phusion High‐Fidelity DNA polymerase (New England BioLabs), cloned into the pJet1.2/blunt vector (ThermoFisher Scientific, Oberhausen, NRW, Germany), and subsequently sequenced. Any mutant lines exhibiting point mutations within the reporter gene constructs were discarded.

### Microscopic analysis

Internal leaf anatomy was assessed on sections sampled from the middle of the second leaf pair (one leaf per plant: three plants per line). Plants were sampled between 09:00 a.m. and 11:00 a.m. and prepared for light and transmission (TEM) microscopy as described by (Khoshravesh *et al*., 2017). The resin blocks that contained leaf material with third vein order were chosen for sectioning. Images for light microscopy were captured on a Zeiss Axiophot microscope equipped with a DP71 Olympus camera and Olympus cellSens image analysis software (Advanced Microscopy Techniques, MA, USA). Images for TEM were captured on a Phillips 201 TEM equipped with an Advantage HR camera system (Advanced Microscopy Techniques, Woburn, MA, USA).

### Mapping by sequencing

Stable M4 mutant lines with intact reporter gene sequences were backcrossed with the corresponding non‐mutagenized reporter gene line. The resulting BC1 plants were selfed and the BC1‐F2 plants were examined for the individual aberrant phenotype. Genomic DNA was isolated from pooled leaf samples of 50–60 BC1‐F2 mutant plants using the DNeasy Plant Maxi Kit (Qiagen, Hilden, Germany). DNA was eluted in 750 μl sterile water in two steps and concentrated to at least 50 ng μl^−1^ by vacuum infiltration.

Sequencing libraries of the pooled mutant DNA as well as of the two‐reporter gene lines were prepared as follows: 1 μg of each DNA sample was sheared with a Covaris S2x system (Covaris, Woburn, MA, USA) to a size of approximately 350 bp. The DNA library was prepared with the TruSeq DNA PCR‐Free LT Library Preparation Kit (Illumina, San Diego, CA, USA) according to the manufacture's manual. The DNA concentrations of the libraries were determined using the KAPA Library Quantification Kit Illumina^®^ platforms (Kapabiosystems, Wilmington, MA, USA).

Paired‐end sequencing (2 × 150 bp) was performed using an Illumina HiSeq3000 system and was carried out by the ‘Genomics and Transcriptomics laboratory’ of the Biologisch‐Medizinisches Forschungszentrum (BMFZ) of Heinrich‐Heine University of Duesseldorf with 80–500‐fold coverage. EMS‐induced mutations potentially responsible for the mutant phenotypes were identified by using SHOREmap v3.0 following the backcross procedure as described (http://bioinfo.mpipz.mpg.de/shoremap/guide.html; Sun and Schneeberger, 2015). Read mapping and SNP calling were performed using SHORE v0.9.3 and Genomemapper v0.4.4.

## Conflicts of Interest

The authors declare no conflicts of interest.

## Supporting information


**Figure S1.** Relative reporter gene signal intensity of all mutant lines.
**Figure S2.** Paradermal sections of the reference line and mutant lines 14, 15, 17, 19 and 20.
**Figure S3.** Structural features of reference and mutant line G‐19
**Figure S4.** Allelic frequencies for mutant line L02 and L03 (Panel a), G21 and G32 (Panel b), and G25 (Panel c).

**Table S1**. Reporter gene expression of 27 EMS mutant lines that were used for light microscopic analysis. +, more signal; −, less signal.
**Table S2**. Oligonucleotides used in this study.
