# Supplementary material for: Reporter‐based forward genetic screen to identify bundle sheath anatomy mutants in A. thaliana
Source: Plant J. 2019 Jan 18;97(5):984–95. doi: 10.1111/tpj.14165 (PMC6850095; doi:10.1111/tpj.14165)
Supplement: Supplementary file 1 — Figure S1. Relative reporter gene signal intensity of all mutant lines. Figure S2. Paradermal sections of the reference line and mutant lines 14, 15, 17, 19 and 20. Figure S3. Structural features of reference and mutant line G‐19 Figure S4. Allelic frequencies for mutant line L02 and L03 (Panel a), G21 and G32 (Panel b), and G25 (Panel c). [file TPJ-97-984-s001.pptx]

## Slide 1
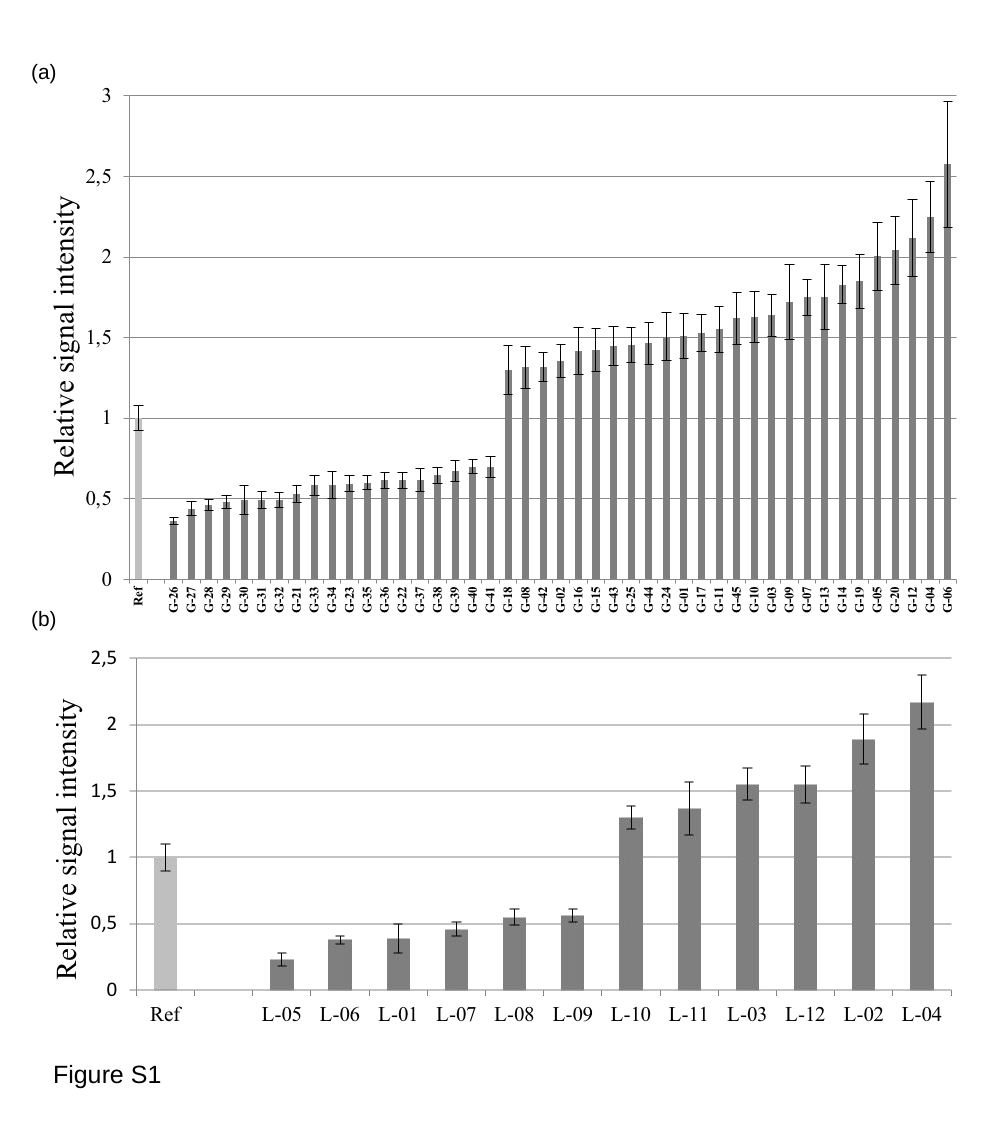

(a)
(b)
(b)
(c)
Figure S1

## Slide 2
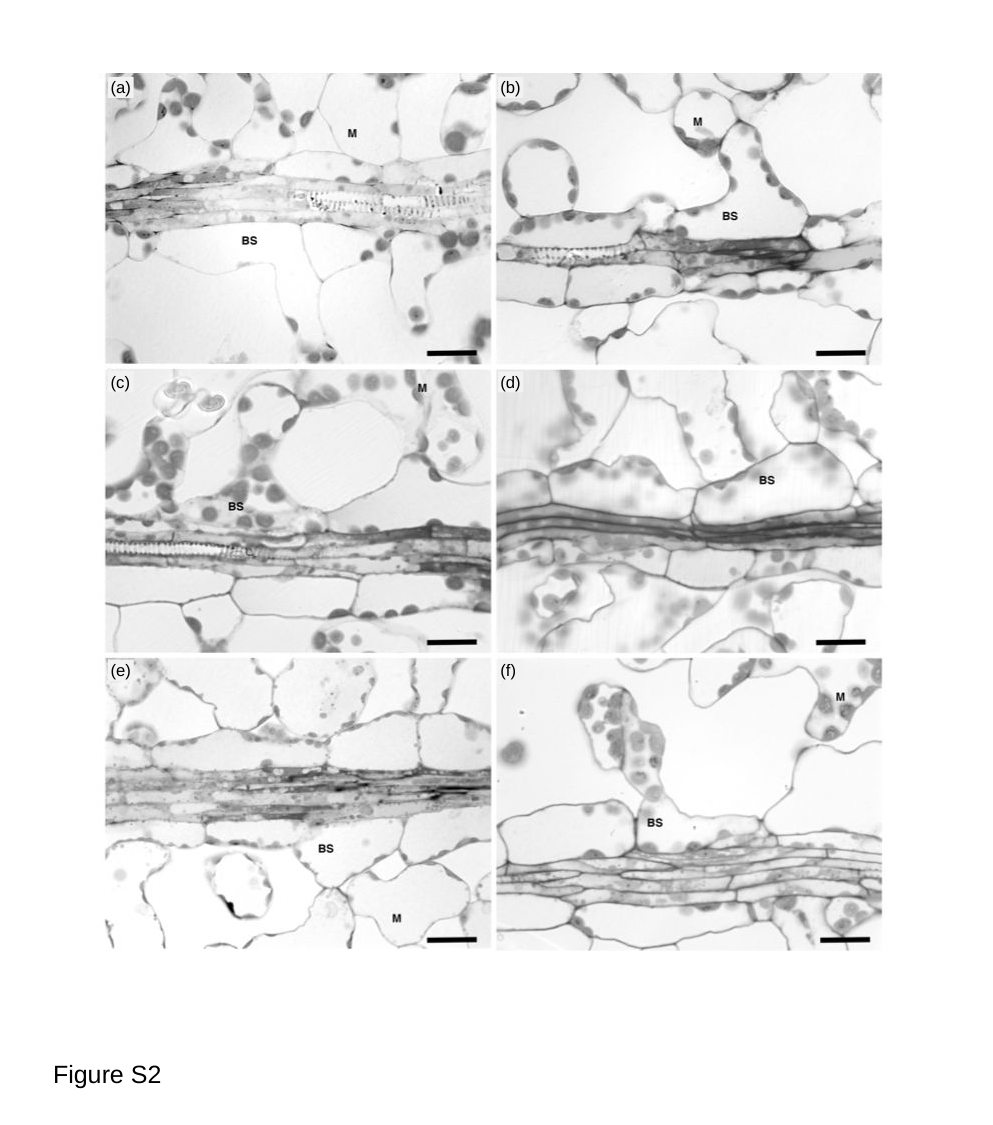

(a)
(b)
(c)
(d)
(e)
(f)
Figure S2

## Slide 3
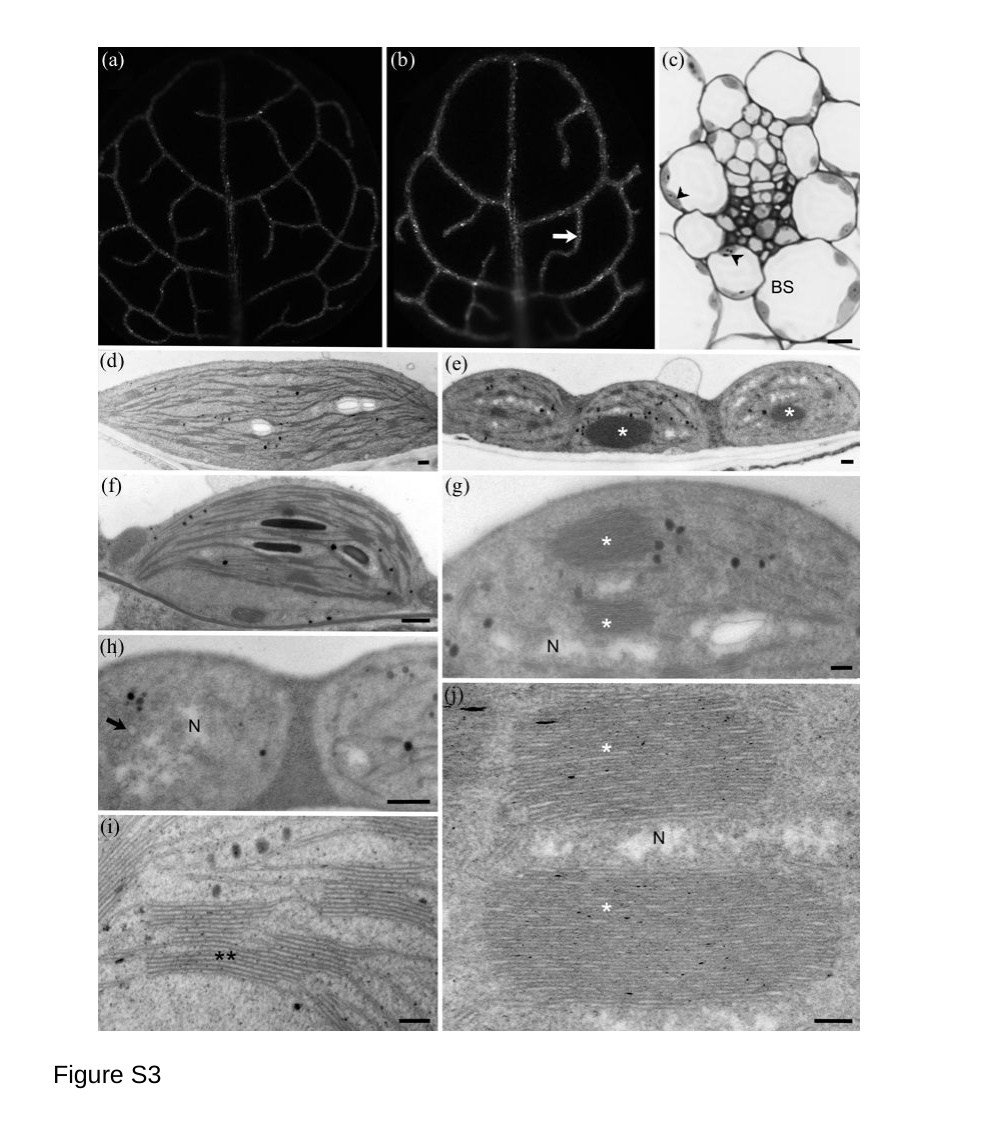

Figure S3

## Slide 4
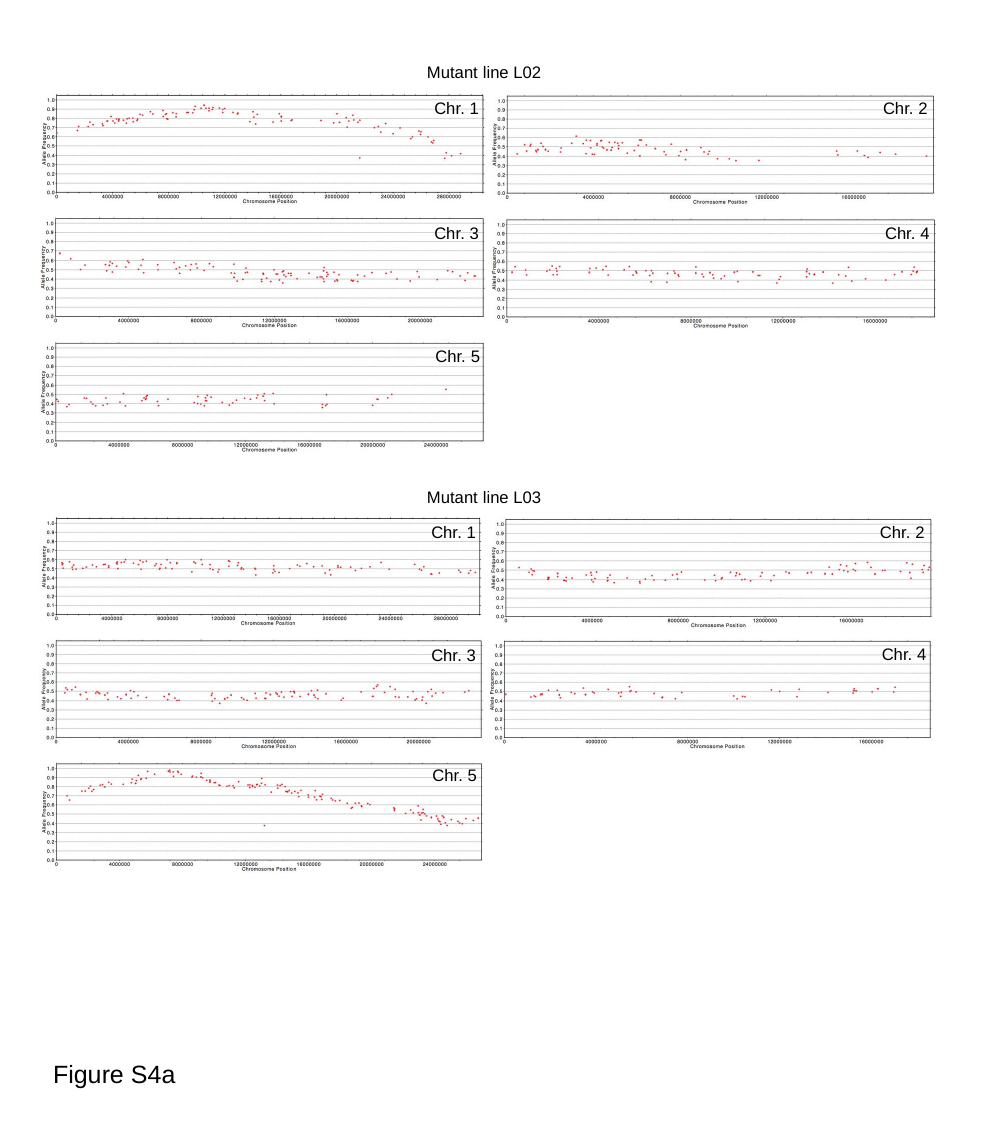

Mutant line L02
Chr. 1
Chr. 2
Chr. 3
Chr. 4
Chr. 5
Mutant line L03
Chr. 1
Chr. 2
Chr. 4
Chr. 3
Chr. 5
Figure S4a

## Slide 5
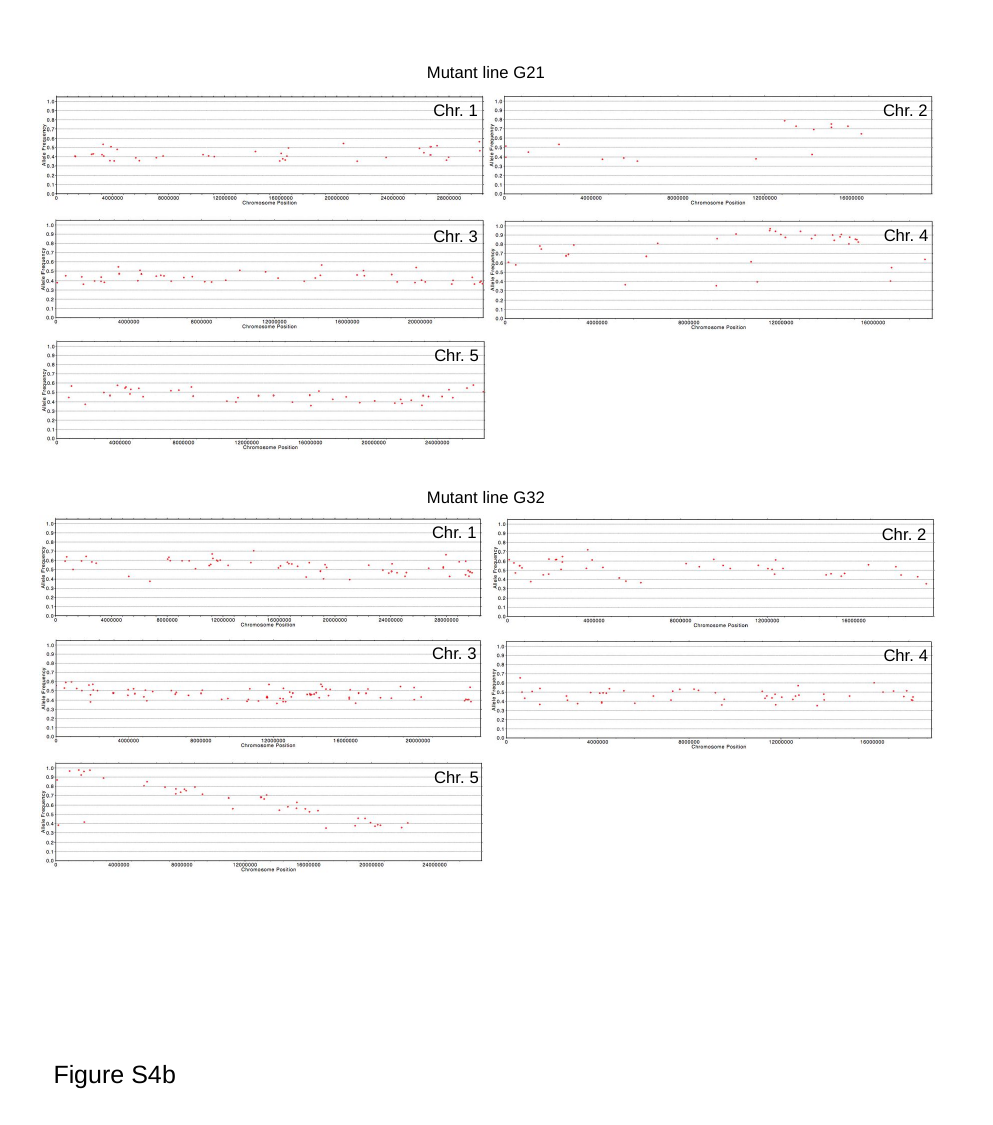

Mutant line G21
Chr. 1
Chr. 2
Chr. 4
Chr. 3
Chr. 5
Mutant line G32
Chr. 1
Chr. 2
Chr. 3
Chr. 4
Chr. 5
Figure S4b

## Slide 6
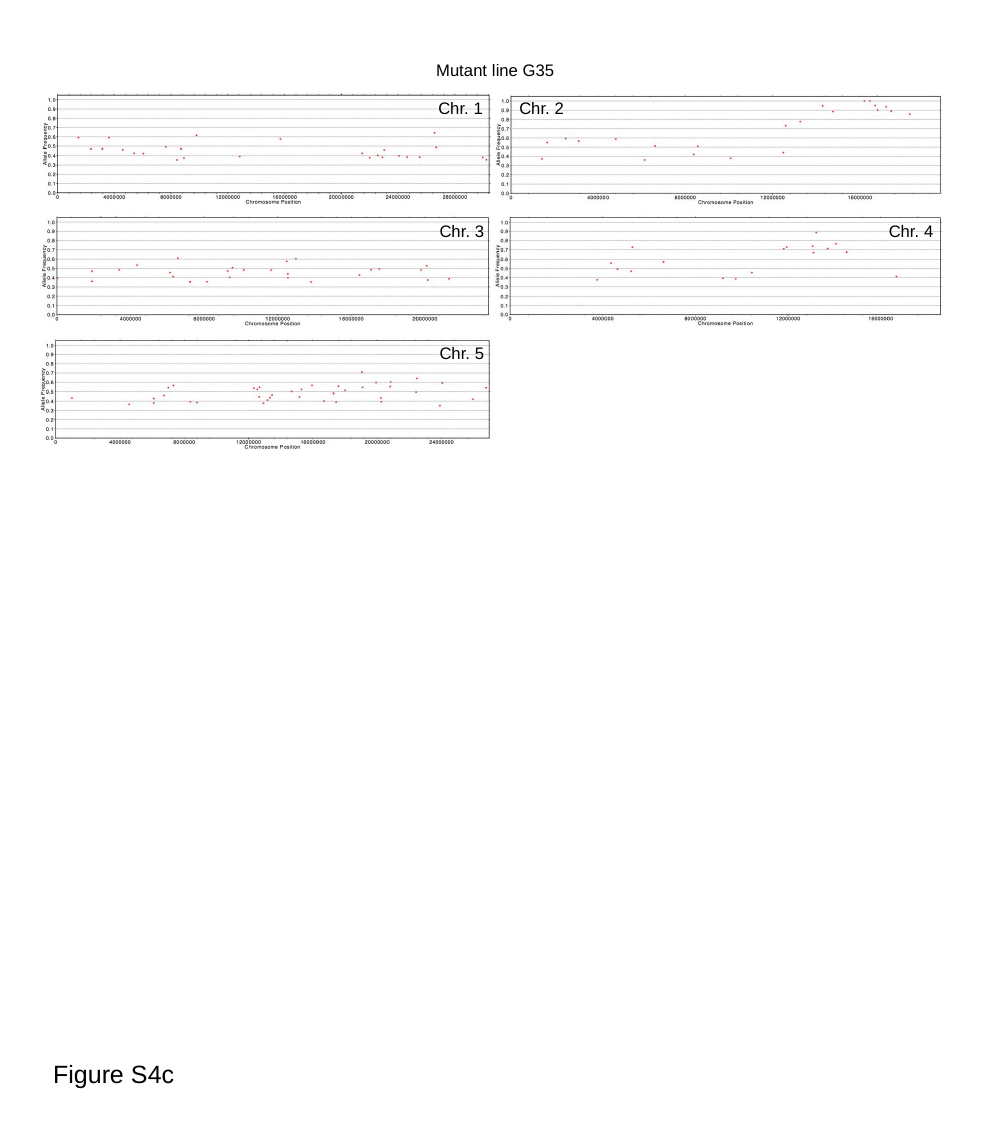

Mutant line G35
Chr. 1
Chr. 2
Chr. 3
Chr. 4
Chr. 5
Figure S4c
