# Supplementary material for: Reporter‐based forward genetic screen to identify bundle sheath anatomy mutants in A. thaliana
Source: Plant J. 2019 Jan 18;97(5):984–95. doi: 10.1111/tpj.14165 (PMC6850095; doi:10.1111/tpj.14165)
Supplement: Supplementary file 2 — Table S1. Reporter gene expression of 27 EMS mutant lines that were used for light microscopic analysis. +, more signal; −, less signal. Table S2. Oligonucleotides used in this study. [file TPJ-97-984-s002.pptx]

## Slide 1
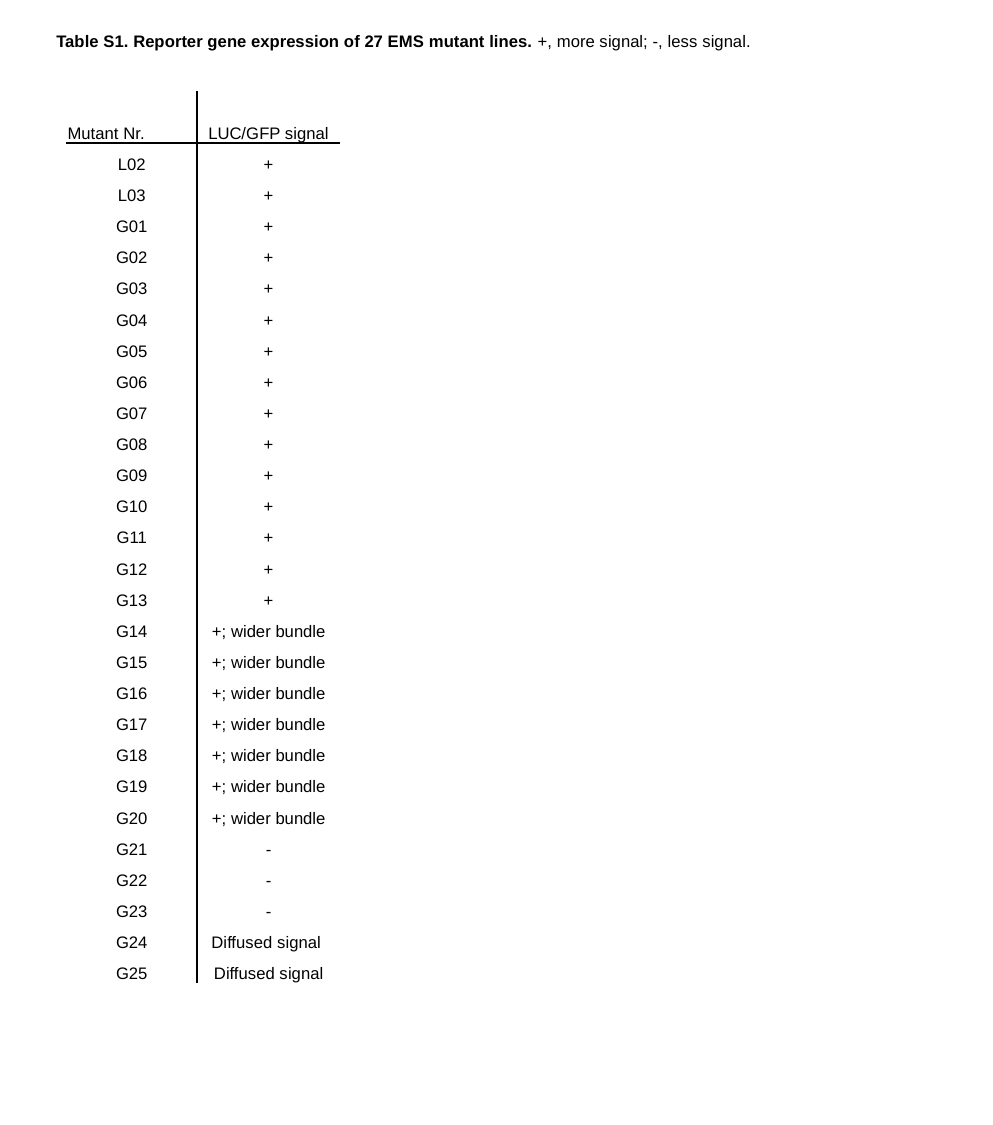

Table S1. Reporter gene expression of 27 EMS mutant lines. +, more signal; -, less signal.
| Mutant Nr. | LUC/GFP signal |
| --- | --- |
| L02 | + |
| L03 | + |
| G01 | + |
| G02 | + |
| G03 | + |
| G04 | + |
| G05 | + |
| G06 | + |
| G07 | + |
| G08 | + |
| G09 | + |
| G10 | + |
| G11 | + |
| G12 | + |
| G13 | + |
| G14 | +; wider bundle |
| G15 | +; wider bundle |
| G16 | +; wider bundle |
| G17 | +; wider bundle |
| G18 | +; wider bundle |
| G19 | +; wider bundle |
| G20 | +; wider bundle |
| G21 | - |
| G22 | - |
| G23 | - |
| G24 | Diffused signal |
| G25 | Diffused signal |

## Slide 2
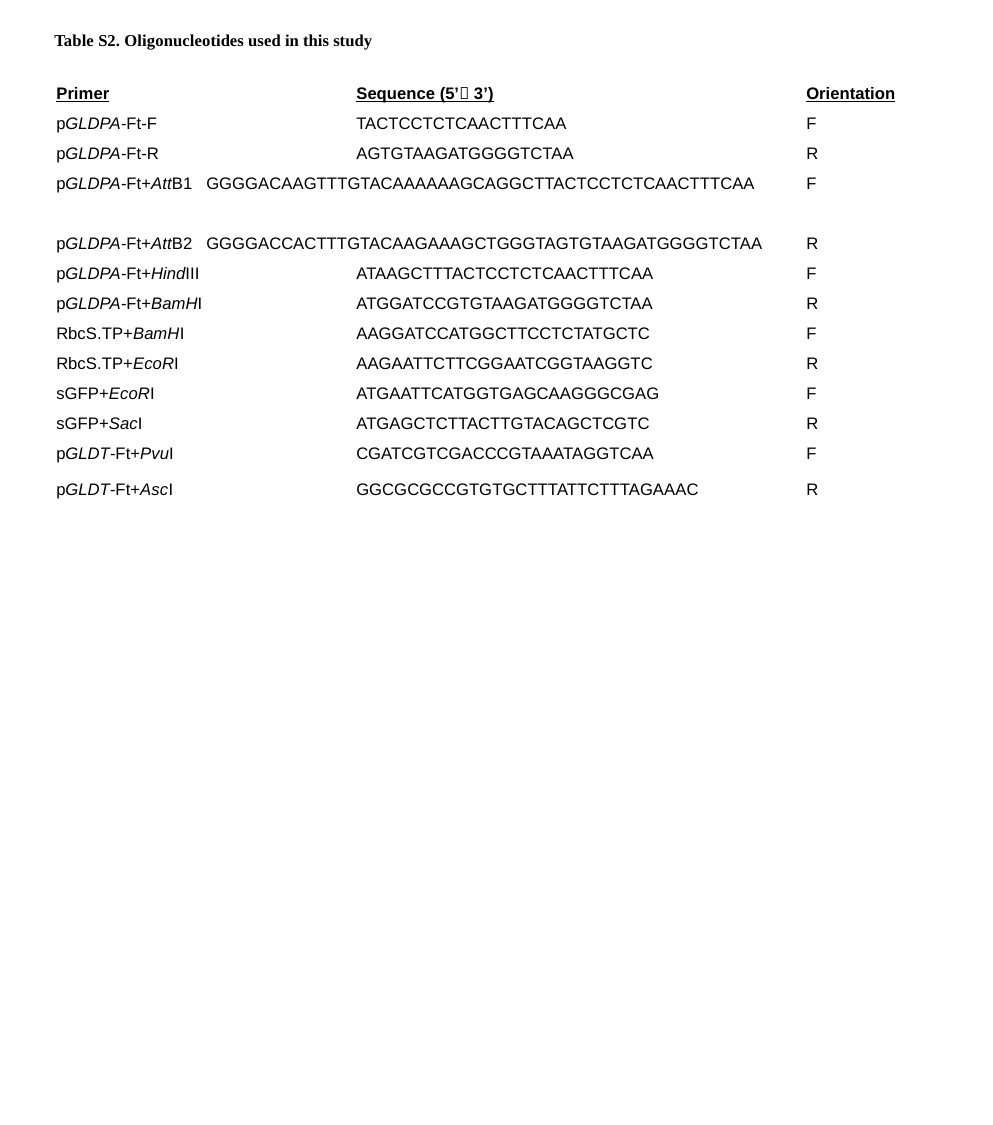

Table S2. Oligonucleotides used in this study
Primer		Sequence (5’ 3’)			Orientation
pGLDPA-Ft-F		TACTCCTCTCAACTTTCAA		F
pGLDPA-Ft-R		AGTGTAAGATGGGGTCTAA		R
pGLDPA-Ft+AttB1	GGGGACAAGTTTGTACAAAAAAGCAGGCTTACTCCTCTCAACTTTCAA	F
pGLDPA-Ft+AttB2	GGGGACCACTTTGTACAAGAAAGCTGGGTAGTGTAAGATGGGGTCTAA	R
pGLDPA-Ft+HindIII		ATAAGCTTTACTCCTCTCAACTTTCAA		F
pGLDPA-Ft+BamHI		ATGGATCCGTGTAAGATGGGGTCTAA		R
RbcS.TP+BamHI		AAGGATCCATGGCTTCCTCTATGCTC		F
RbcS.TP+EcoRI		AAGAATTCTTCGGAATCGGTAAGGTC		R
sGFP+EcoRI		ATGAATTCATGGTGAGCAAGGGCGAG	F
sGFP+SacI		ATGAGCTCTTACTTGTACAGCTCGTC		R
pGLDT-Ft+PvuI		CGATCGTCGACCCGTAAATAGGTCAA		F
pGLDT-Ft+AscI		GGCGCGCCGTGTGCTTTATTCTTTAGAAAC	R
